# Supplementary figures and images for: Comparative genome analysis indicates high evolutionary potential of pathogenicity genes in Colletotrichum tanaceti
Source: PLoS One. 2019 May 31;14(5):e0212248. doi: 10.1371/journal.pone.0212248 (PMC6544218; doi:10.1371/journal.pone.0212248)

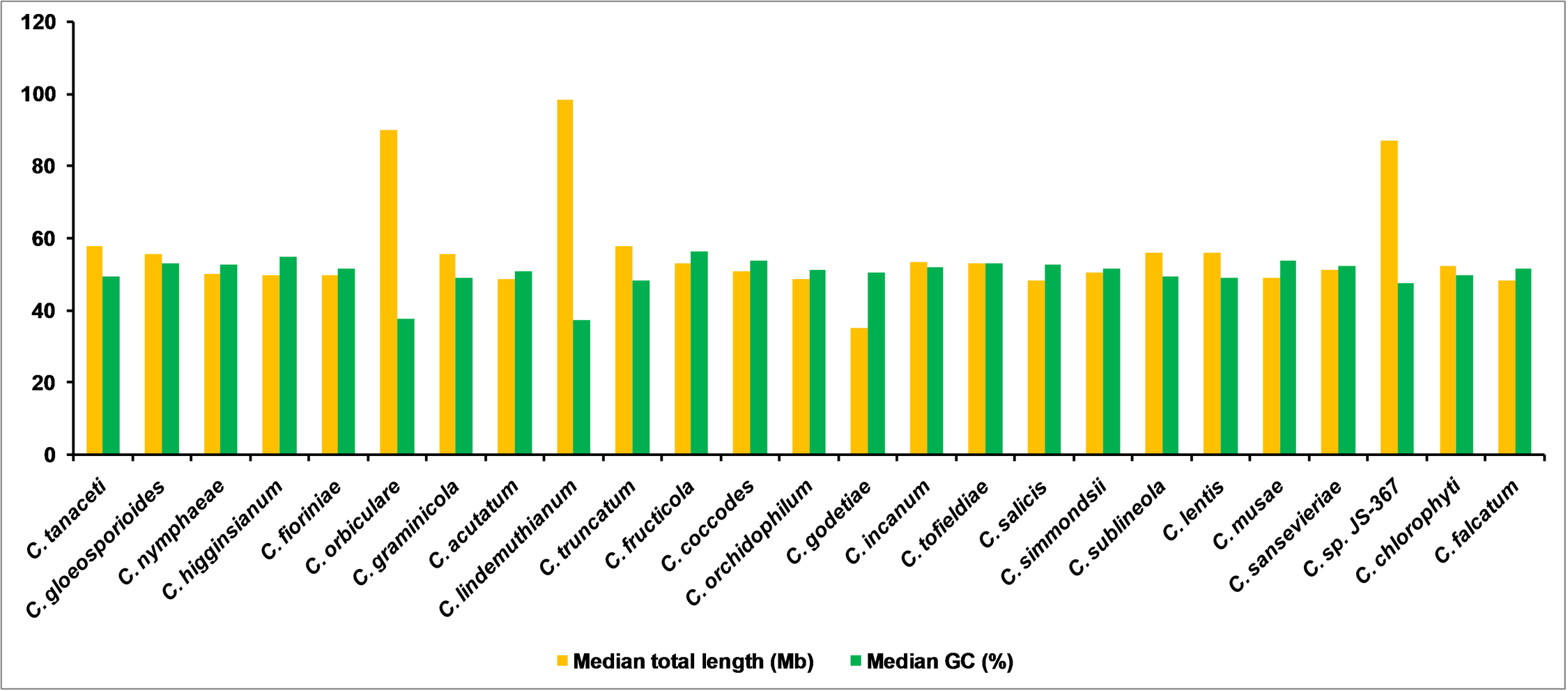

Supplement: S1 Fig — (TIF) [file pone.0212248.s023.tif]

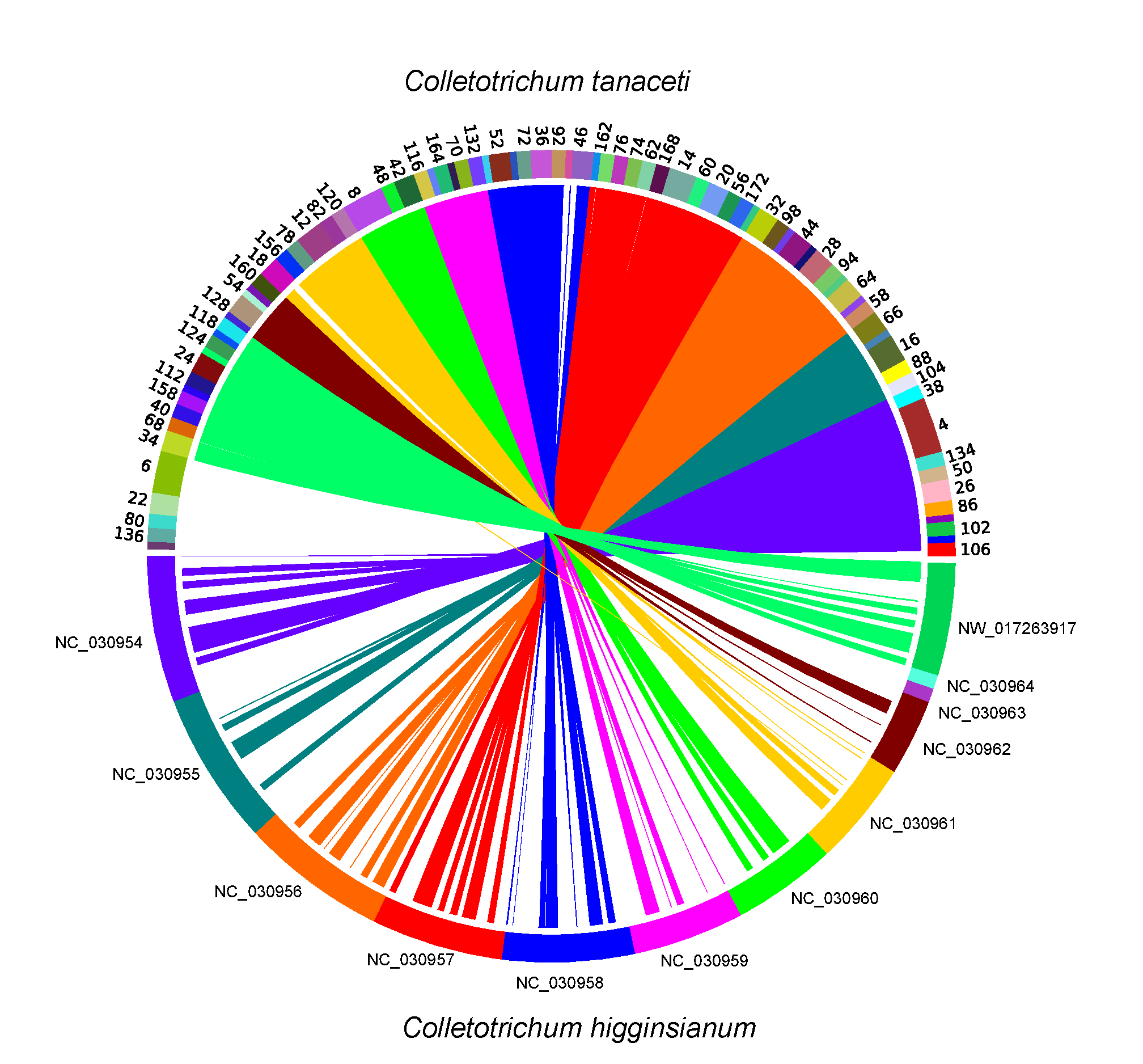

Supplement: S2 Fig — (TIF) [file pone.0212248.s024.tif]

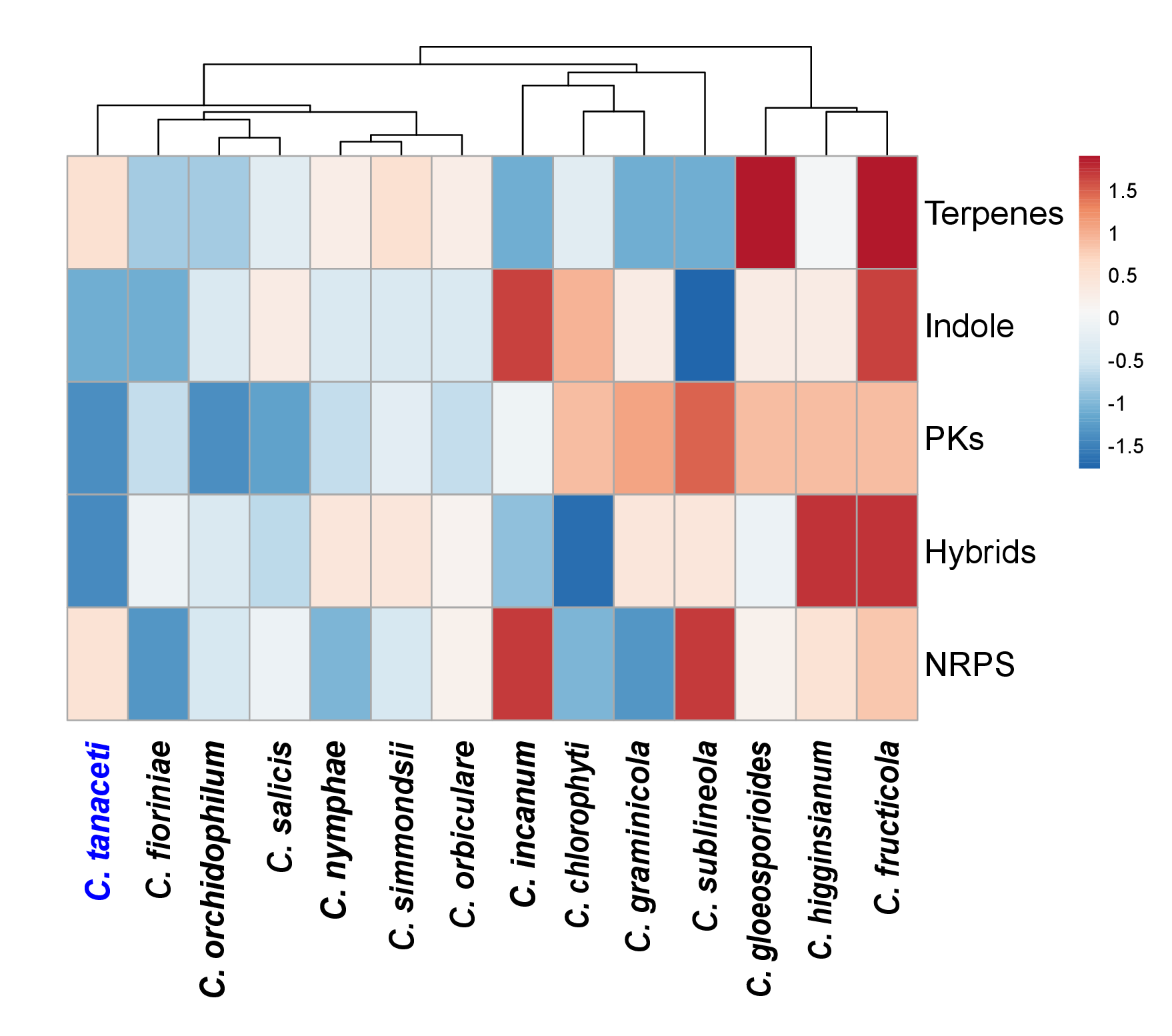

Supplement: S3 Fig — The number of genes in each gene category is normalized using unit variance scaling. Overrepresented and underrepresented types of secondary metabolite gene clusters are represented in red to orange and blue respectively as fold standard deviations above and below the mean. (TIF) [file pone.0212248.s025.tif]

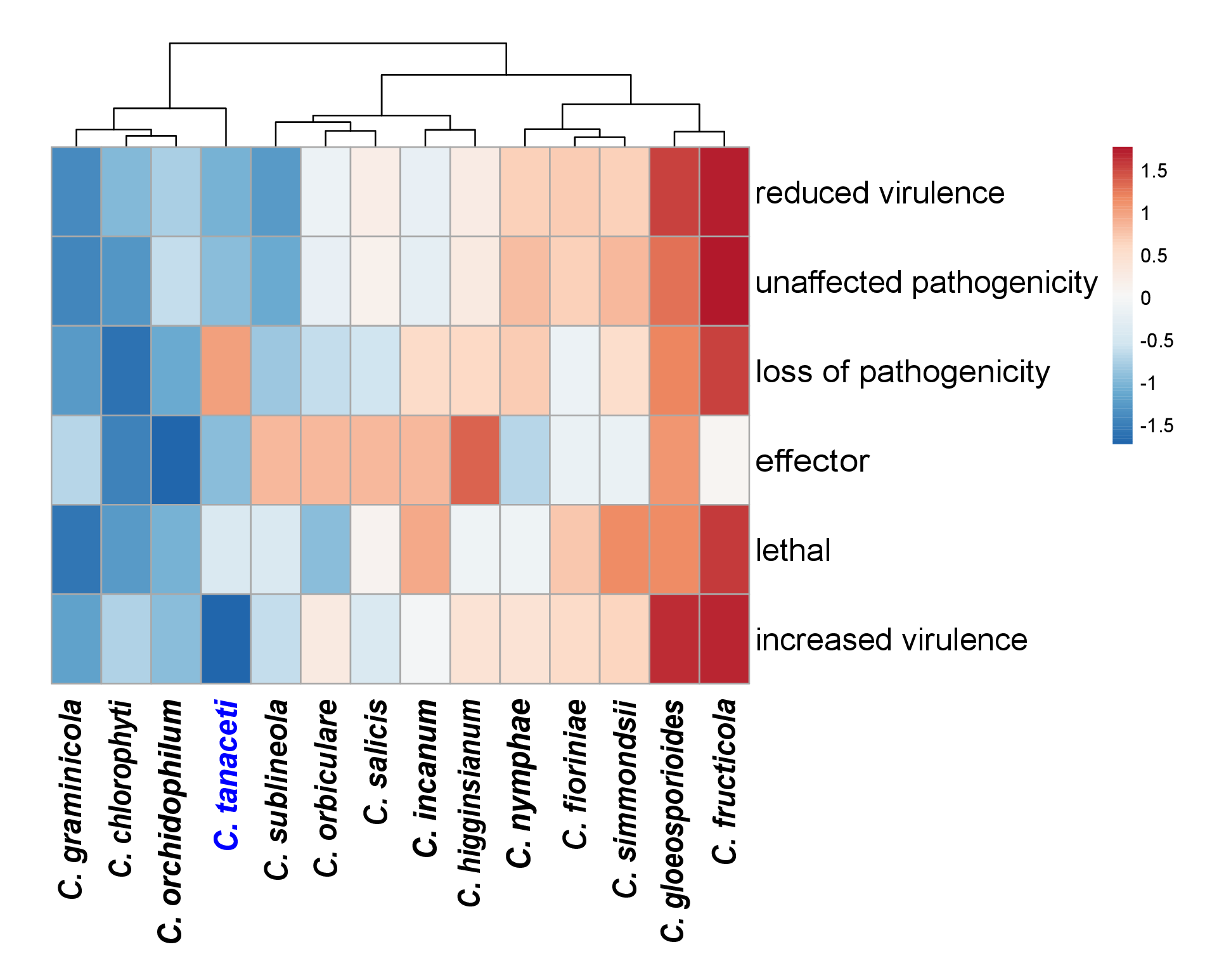

Supplement: S4 Fig — The number of genes in each PHI category is normalized using unit variance scaling. Overrepresented and underrepresented gene categories are represented in red to orange and blue respectively as fold standard deviations above and below the mean. (TIF) [file pone.0212248.s026.tif]
